# Health and medical care for refugees: design and evaluation of a multidisciplinary clinical elective for medical students

**DOI:** 10.3205/zma001435

**Published:** 2021-02-15

**Authors:** Sandra Ziegler, Katharina Wahedi, Mariella Stiller, Rosa Jahn, Cornelia Straßner, Simon Schwill, Kayvan Bozorgmehr

**Affiliations:** 1 Heidelberg University Hospital, Section Health Equity Studies & Migration, Department of General Medicine and Health Services Research, Heidelberg, Germany; 2 Bielefeld University, School of Public Health, Department of Population Medicine and Health Services Research, Bielefeld, Germany

**Keywords:** refugees, medical students, cultural diversity, people in situations of vulnerability, minority health, teaching, medical education, program development, program evaluation

## Abstract

**Objective: **Caring for refugee patients places special demands on health professionals. To date, medical students in Germany have rarely been systematically prepared for these challenges. This article reports on the development, conceptualisation, implementation, evaluation, and relevance of a multidisciplinary elective for medical students in the clinical study phase.

**Methodology: **The course content was developed based on a needs-assessment among medical students and in cooperation with medical colleagues working in the field of refugee care. The course consisted of a seminar with medical, legal, administrative and socio-cultural learning content as well as a field placement in the medical outpatient clinic of the local reception centre for asylum seekers, which was accompanied by a systematic reflection process. The evaluation concept contained qualitative and quantitative elements.

**Results: **123 students completed the elective over six semesters (summer 2016 through to winter 2018). It was continuously evaluated and further developed. The students reported learning progress mainly in the following areas: Legal foundations of the asylum procedure and health care entitlements for asylum seekers; multi-perspectivity through multidisciplinarity as well as professional, ethical, interpersonal, and political insights gained through the practical experience.

**Summary:** To prepare students for the complex challenges to be faced in medical care for refugees, a structured, multidisciplinary teaching programme, which combines theory, practice and reflection helps to foster insights into the many facets of this field of activity. The questions students brought to the seminar, the course contents and evaluation results are intended to inspire the design and implementation of similar courses at other universities.

## Zusammenfassung

**Zielsetzung: **Die medizinische Versorgung Geflüchteter stellt besondere Anforderungen an Gesundheitsfachkräfte. Medizinstudierende in Deutschland werden bisher kaum systematisch auf diese vorbereitet. Im Beitrag wird über die Entwicklung, Konzeption, Umsetzung, Evaluation und Bedeutung eines multidisziplinären Wahlfachs für Medizinstudierende im klinischen Studienabschnitt berichtet.

**Methodik: **Die Entwicklung der Kursinhalte erfolgte auf Grundlage von Bedarfsanalysen bei Medizinstudierenden und in Kooperation mit in der Flüchtlingsversorgung tätigen ärztlichen Kollegen. Die Lehrveranstaltung bestand aus einem Seminar mit medizinischen, juristischen, administrativen und sozio-kulturellen Lerninhalten sowie einer systematisch reflektierten Hospitation in der medizinischen Ambulanz des lokalen Ankunftszentrums für Asylsuchende. Das Evaluationskonzept enthielt qualitative und quantitative Elemente.

**Ergebnisse: **Ab dem Sommersemester 2016 bis einschließlich Wintersemester 2018 absolvierten 132 Studierende das Wahlfach. Es wurde fortlaufend evaluiert und weiterentwickelt. Mehrheitlich werden vor allem Lernfortschritte in folgenden Bereichen berichtet: Rechtliche Grundlagen des Asylverfahrens und der Versorgung; Mehrperspektivität durch Multidisziplinarität sowie Erkenntnisse durch die Praxiserfahrung im fachprofessionellen, berufsethischen, zwischenmenschlichen und politischen Bereich.

**Zusammenfassung: **Mittels eines strukturierten, multidisziplinären Lehrangebotes, welches Theorie-, Praxis- und Reflexionselemente verknüpft, kann Studierenden ein Einblick in die vielen Facetten der medizinischen Versorgung Geflüchteter ermöglicht werden, um sie auf die komplexen Herausforderungen dieses Handlungsfeldes vorzubereiten. Die Studierendenfragen, Kursinhalte und Auswertungsergebnisse können Anregung für die Implementierung ähnlicher Lehrveranstaltungen geben.

## 1. Introduction

Due to increased flight migration in recent years, the diversity of the patient population in Germany has additionally increased. Between 2014 and the end of 2016, the number of people seeking protection rose by 113% to 1.6 million [1]. In Heidelberg, a former US Army housing estate, the Patrick-Henry-Village (PHV), was set up as emergency accommodation at the end of 2014. At times, up to 8000 refugees have been accommodated there [2], [3]. The local clinics recorded an increased patient influx, mostly requiring primary medical care [4], [5]. To meet these needs, relieve the clinics and improve the medical care of the refugees, an outpatient clinic was set up on the premises of the PHV offering care by professionals from five medical disciplines. Medical students were also actively involved in this complex care setting [3]. In order to cope with the legal, medical, organisational, socio-cultural and ethical challenges they faced (cf. [6]), the students asked for structured learning and reflection opportunities. 

In the outpatient and inpatient care of refugees, staff are regularly confronted not only with language barriers, but also with perceptions and concepts of the body, health, illness and treatment that may differ from their own ([7], p. 109f; [8], p. 71-79). Furthermore the patients’ diverse individual experiences during the pre-, peri- and post-migration phase can be relevant to their illness, its course and treatment [9], [10]. Their access to medical services may also be restricted depending on their residence status and duration of stay (cf. [11], [12], [13]). In addition, their living conditions (e.g. in shared accommodation) and precarious socio-economic situation must be taken into account [14]. Competent medical personnel can therefore overcome “socially and culturally constructed” differences ([15] p. 168), has developed an awareness of the context of these patients and successfully copes with everyday practical demands.

However, doctors-to-be are often insufficiently prepared through their studies [16] for the communicative, structural and medical requirements of medical care for refugees (cf. [17], [18], [19], [20], [21]). This gap in medical education is currently being addressed by local teaching initiatives (cf. [22], [23], [24], [25], [26]. Recurring features of these courses are intercultural topics, multi-professional and -disciplinary approaches and different types of practical orientation. Course materials are rarely made publicly available [27]; general training needs are stated, but rarely specified in relation to the specific students who are taught. Oftentimes singular aspects are addressed, such as theoretical principles [28]; or the standardised measurement of effects [24], or there is only a brief overview of the course design (e.g. in the case of [23], [29]), which limits the possibility to replicate it. 

The Section Health Equity Studies & Migration of the Department of General Medicine and Health Services Research at the Heidelberg University Hospital, developed a multidisciplinary elective on “Health and Medical Care for Refugees”. Within this article we want to

1. present the basic features of the course concept and implementation in a way that can serve as an example for the design of similar courses at other universities; and
2. show survey and evaluation results that contributed to the assessment of the training needs and the competence acquisition of students and served to further develop the course. These objectives refer to our goal
3. to demonstrate the relevance of such a course for a modern, diversity-sensitive medical education.

## 2. Learning objectives and course concept

Our programme combined theoretical, practical and research elements. Students were given the opportunity to acquire specific professional skills for refugee care and to gain insights into care and research practice (see learning objectives, table 1 (Tab. 1) and course program as well as lecturers’ expertise, table 2 (Tab. 2)). Diversity sensitivity requires the cultivation of an interdisciplinary view "beyond the horizon" of medical issues [30] p. 14; cf. [31] and NKLM, e.g. ID 11.4.3.1 14.c.5.2.1/4/5 [http://www.nklm.de]. It requires an open, curious and reflective attitude towards oneself and one's counterpart, taking into account individual (cf. [32], p. 206, [33], [34]), collective, lifeworld and socio-cultural contexts. Reflection on social attribution and differentiation practices as well as their interdependencies has to be fostered (cf. [35], [36], [37]). Students should also be able to identify discriminatory practices (cf. [32], [203], [38], p. 544). Diversity competence understood in this way must be supplemented by structural competence [39], that is a critical glance at society and the complex structures and power relations in which the field of medical care for refugees is embedded. 

**Figure 1:**
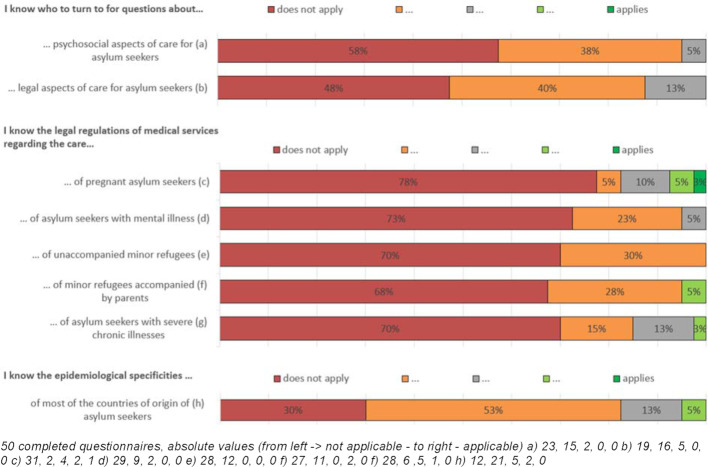
Knowledge level relevant to refugee health care (cohort 1+2)

The initial curriculum was designed according to the practical requirements of everyday medical practice in the outpatient clinic, as reported by student and medical colleagues. Scientific literature, for example [38], [40], [41], [42], [43], [44], [45], [46], [47] and similar initiatives [22], [25], [26], [48] provided further inspiration. In order to develop and improve the curriculum and to adapt it to the knowledge and interests of the students, we conducted a one-page survey regarding the existing knowledge in the first two cohorts (see figure 1 (Fig. 1) below). Starting from the second cohort, we also collected student expectations. From the third cohort onwards, this was transformed into a collection of concrete questions that the students wanted to have answered by the end of the semester (for examples of specific student questions, see attachment 1 ). 

## 3. Main features of the course

### 3.1. Learner centred and multidisciplinary program

To provide a comprehensive overview of relevant topics, we involved experts and practitioners from various disciplines (see table 2 (Tab. 2)). Students attended a total of 24 hours of class time. Each three-hour session included two thematic units, each composed of 60-70 minutes presentation time and 15-35 minutes for questions, discussion and evaluation. 

We offered 24 course places per semester. The students' questions of the respective cohort (see attachment 1 ) were arranged thematically and sent out to each lecturer in advance of their lecture, with the request to answer the questions assigned to their expertise. These questions were also posted in the seminar room on the day of the course. The course coordinator – who is the first author – was present at all sessions and ensured that the questions were addressed. She also managed the administrative and content coordination. Moderation by the course coordinator ensured a diversity-sensitive and discrimination-critical perspective and helped establish connections between individual topics, fostering interconnections of perspectives from multi-towards inter-disciplinarity. To this end we also invited both lecturers of a session to be present during the entire session and engage in a joint discussion with students and their colleague. 

Two sessions during the semester were designed by the students themselves. They were invited to share their experiences during the field placement in the outpatient clinic of the reception centre and to reflect on the social context, structures and processes in which refugee care is embedded [49]. The students were asked to prepare a question or topic of their choosing and share a case report or bring a scientific or journalistic text, which was to be discussed in a breakout group. Topics from students included, for example, the consequences of the “interpretability” of the asylum seekers benefit act (which regulates the access to care for asylum seekers in Germany) or the potential political motivation for refugee accommodation often being located in remote settings or the outskirts of towns or cities, with students critically discussing the effects on integration and health (for further examples of discussion topics, see attachment 2 ) 

#### 3.2. Practical experience: Community engagement and ethnography

All participants spent two days in the local outpatient clinic for refugees – compulsory in general medicine and optional in paediatrics, gynaecology/obstetrics, or psychosocial medicine. Involvement in this real world context makes the political, normative and social aspects of medicine tangible [46], [47]. By providing the opportunity to gain experiences in caring for a structurally disadvantaged population (in the health system and society) [50], p. 1939 we intended to raise awareness of special needs and vulnerabilities and to make students aware of their social responsibility (or accountability), especially with regard to health equity [51], p. 8. 

In order to facilitate the process of experience-based and exploratory learning [52], [53] the field experiences were reflected upon within seminar discussion sessions as well as through a 8-15 page written report. One task of the report was an ethnographic observation exercise including fieldnotes [54], [55], while another was the reflection of a medical and a structural, socio-cultural or psycho-social aspect of the care situation. Furthermore, students were asked to give a “best practice” example of refugee care as well as a practice worthy of being criticised. They should describe professional competences shown in the former and behavioural alternatives for the latter medical encounter. Finally, they were invited to identify their own knowledge and training deficits as well as future strategies for personal and professional development (for guidelines to the field report, see attachment 3 ). Additional to a grade, the learners received detailed, individual written feedback on their report. 

## 4. Ongoing development of the course: Evaluation concept and methodology

Student expectations, questions (see chapter 2 and attachment 1 ) and feedback continuously informed the course development. In addition to individual evaluations of each session and lecturer (which we do not report on here) a final evaluation was carried out at the end of each semester, starting in winter semester 2016/17. The course was assessed by means of an anonymous, written evaluation sheet (see attachment 4 ). We asked students to provide a grade for the course as well as a self-assessment of their knowledge and competence gain through the course components on five-point Likert scales (“does not apply” to “applies”). On four-point scales, students reported on whether the course had been worthwhile for them (“not applicable at all” to “fully applicable”) and how satisfied they were with the placement in the respective medical disciplines of the outpatient clinic (“very dissatisfied” to “very satisfied”). The compiled data of four cohorts (winter semester 2016/17 to summer semester 2018) was analysed descriptively using a spreadsheet program. 

Students could also provide feedback on the field placement and the written and oral reflection modules in additional free-text boxes. Starting in summer semester 2017 we also asked in an open-ended manner about the learning outcome and take home messages that were personally considered most important. The qualitative feedback was digitalised and underwent a category-based, inductive content analysis. We identified frequently mentioned topic areas for each evaluation question [56], p. 78, [57] and developed further categories to which the remaining data material was assigned. Topics mentioned only once were compiled separately and are omitted here (cf. [58]). 

## 5. Results

The course was implemented as a clinical elective at the Medical Faculty of Heidelberg University. It was later established as a permanent component of the “Global Health Track” of the Faculty. A total of 132 students completed the course during a period of six semesters over three years, from summer semester 2016 to winter semester 2018/19. After the course places were allocated to students, an average of 6.4 students had to be put on a waiting list and were given priority admission in the following semester. 

### 5.1. Survey of existing knowledge and student questions

Our survey of the educational level (n=50 participants of the first two cohorts: winter semester 2016/17, summer semester 2017) showed that basic refugee health care related knowledge was missing. For example, more than two thirds of the students were unfamiliar with its legal regulations (Self-assessment of knowledge level relevant to refugee health care, see figure 1 (Fig. 1)). 

Some students (21%) stated experience in working with refugees, for example involvement in setting up the outpatient clinic or completing a clinical traineeship there. The majority lacked experiences with refugees and had numerous questions about their care (see attachment 1), which our multidisciplinary team was able to answer or address. 

#### 5.2. Evaluation

##### 5.2.1. Assessment of competence acquisition and learning outcomes

**Figure 2:**
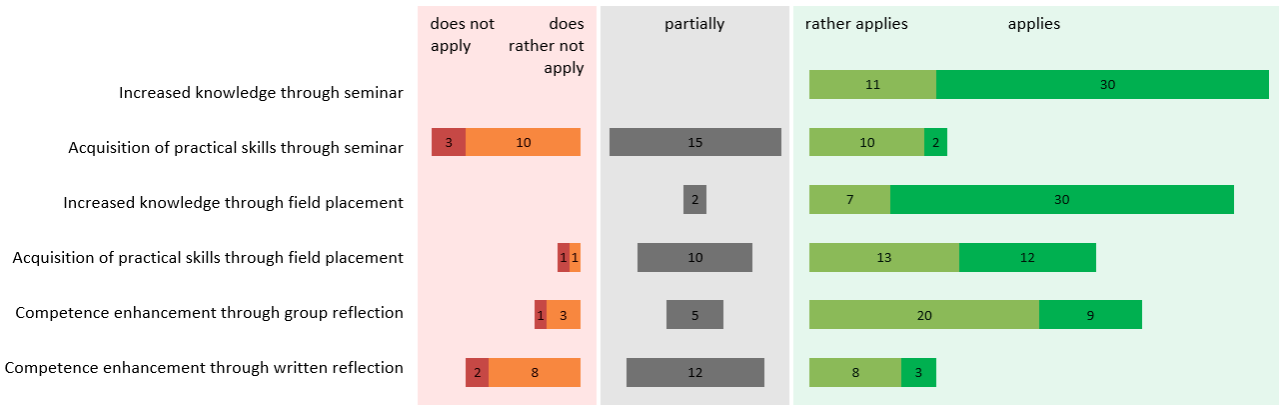
Self-assessment of improved knowledge and competencies through the seminar components

50 out of 91 students took part in the detailed written course evaluation (winter semester 2016/17 to summer semester 2018). The aggregated data (of the 5-point- scales) shows that students agreed that their knowledge about the health care of refugees was extended by participating in the seminar (*M*=4.7; *SD*=0.9). The field placement was on average evaluated as positive for knowledge gain (*M*=4.7; *SD*=0.9). There was a wider distribution of opinions regarding the practical skills gain through the course (*M*=2.95 and *med*=3, i.e. “partially”, *SD*=1.1) and field placement (*M*=3.9; *med*=4 at “rather applies”; *SD*=1.2) (see figure 2 (Fig. 2)). 

The qualitative results demonstrated that student learning progress was largely in line with our teaching objectives. Over all semesters analysed, the most frequently reported knowledge gain (in ascending order) related to legal aspects of the asylum procedure, acquired multi-perspectivity through multidisciplinarity, and important insights through practical experience in the outpatient clinic. Here students reported, for example, that they could empathise with refugees or understand and critically reflect on situations better with reference to the course content and discussions (for a compilation of student statements about their most important take-home-messages, see attachment 5 ). Some statements indicated that student expectations and questions were modified throughout the course: exoticisation and culturalisation could be replaced by a realistic assessment of everyday tasks in refugee medicine. Others reported to have become more aware of how personal, professional, national and global contexts affect medical practice. This was nicely summed up by one student who stated that, as a doctor, one has not only medical but also social and political responsibility. 

##### 5.2.2. Evaluation of the field placement

Each student completed an internship in two disciplines of the refugee outpatient clinic. The majority of students stated that they were “rather satisfied” or “very satisfied” with this field placement (89% of cases), with variations between disciplines (see table 3 (Tab. 3)). 

Analysis of larger clusters of (critical) free-text feedback identified challenges in the following three areas:

1. organisational obstacles (e.g. waiting times at security checkpoints),
2. the little predefined role on site (observer vs. "errand boy" or self-responsible medical assistant) and
3. the desire to extend the period of practical training.

Praise was given for the “nice atmosphere on site” (4x), where “despite the high work-load”, health professionals assigned “a high amount of time” to the student. Some course participants also used the feedback opportunity to assess the quality of care and problematised the way some providers dealt with patients (4x). The lack of communication between the numerous providers on site and the fragmented local documentation procedure were also discussed critically.

#### 5.3. Evaluation of the reflexive seminar elements (discussion groups and report)

The majority of students agreed that competencies had been acquired through the structured, oral reflection of their experiences in the refugee outpatient clinic during the course (rather applies: 53%, applies: 24%) (*M*=3.9 out of 5; *SD*=1.1). Whether the written reflection also contributed to the acquisition of competencies many could not say with certainty (*M*=3.1, *SD*=1.2). With regard to the ethnographic exercise, methodological uncertainties and obstacles in the field were repeatedly expressed. The number of positive and negative comments on the exercise was balanced. Some found that the exercise trained perception and reflection and enabled “completely new insights”, while others referred to it as not very profitable and too much effort. 

#### 5.4. Overall assessment

Students were asked to grade the course according to the grading system used at German schools (from 1-6, 1 meaning “very good”, 6 meaning “insufficient”). The average grade given to the course by the students was 1.74 (*SD*=0.6). For the statement that the course participation was worthwhile 33 of 50 students “fully” agreed and 17 “rather” agreed (*M*=3.7; *SD*=0.7).

#### 5.5. Limitations 

All students who attended the last seminar session of each semester took part in the evaluation. The response rate was 55%, which can be largely explained because the number of participants was typically reduced in this last session due to ongoing exams in other courses. Our evaluation primarily served to improve the course; in order to increase the scientific significance of the results, further adjustments would be necessary. For example, student competence acquisition can also be observed during thematic discussions in break-out groups and within the final written report. After appropriate operationalisation both could be profitably included in the evaluation (cf. e.g. in [22]). At the end of the semester student questions (see attachment 1 ) were collectively compared with the content of the curriculum. We have access to written self-assessments in the evaluation and the final reports, which capture acquired (see, for example, attachment 5 ) or lacking skills and knowledge (according to point 3 of the field report, attachment 3 ) in different competence dimensions. In the future, this qualitative data collected before and after the course could be matched at the level of individual students. We could also develop a long term pre-post evaluation (cf. [59]) with standardised evaluation instruments. This would increase the comparability over time and with other studies. To this end, we could aim at a pseudonymisation of the evaluation-survey, since our anonymisation – without any socio-demographic data collection – reduces analytical possibilities beyond mere description. 

Possible limitations in the implementation of a teaching project like this one lie in financial, time and personnel resources as well as the access to local networks. Only in close cooperation with local health care institutions for refugees as well as responsible (government) authorities, can a field placement be made possible. Another prerequisite for the design and implementation of the course is a committed coordination team with the relevant expertise in the subject area. The assessment of the relevance of the topic by funding organisations and faculties is another secondary factor upon which implementation is dependent. Personnel must invest a significant amount of time in concept development, implementation, organisation, student support and continuous evaluation and funds must be available for invited experts. After the course has been successfully run for six semesters and was implemented in the teaching structures of the Heidelberg Medical Faculty, its funding was no longer secured, at the end of the reported period. It would therefore be advisable that similar courses or at least content should leave the optional frame (cf. [30], p. 12) and be integrated into the structures of the regular medical curriculum to ensure that future doctors are prepared for the challenges of modern, diverse societies. 

## 6. Discussion

There are large gaps in medical education to provide effective, high quality health care to refugees. This was not only shown by our preliminary survey, but also by a -larger study with 523 medical students in Germany [16], p. 168ff. In order to prepare students for the challenges of caring for this heterogeneous population, we implemented a multidisciplinary elective in refugee health care at the medical faculty in Heidelberg. The learning successes reported by our students illustrate the educational impact of the innovative course and its relevance for modern medical education. We presented our concept, the curriculum – including practical and reflexive modules – as well as selected student feedback, to provide relevant material for similar initiatives in other educational institutions and settings. The evaluation results demonstrate that our course was able to adequately address student questions and expectations and that the learning outcome were predominantly in line with the objectives or related to the presented content. The practical field experience in the local refugee outpatient clinic was of great importance for student learning.

In order to become competent professionals in this field, prospective doctors need basic medical and communicative knowledge to cope with the everyday tasks of refugee care. In addition, they should learn to approach each patient in an open and curious manner, in order to find out about and understand the meaning and impact of socio-cultural belonging structures for the individual patient and for their understanding of the disease and its treatment [32], [60] without discriminating in professional practice according to ascribed characteristics (cf. [61]) regarding, for example, religion, ethnicity, nationality or social position (cf. Declaration of Geneva [62]). 

The health care personnel should be aware of legal frameworks, care structures and processes, and learn about involved actors as well as motives and interests of the various stakeholders. The medical role of being a responsible health advocate (“Gesundheitsfürsprecher”, “Verantwortungsträger”) is particularly important to reduce disparities and improve the quality of care for refugees (cf. NKLM [http://www.nklm.de] pp. 17, 52, 54f). To become such advocates, and put the patients well-being first [63], doctors have to be able to realise when interpretative patterns, from other sectors of the society – in the case of asylum seekers of the political, legal and administrative system – penetrate the health system and with it the common logics of medical action. 

According to §2 (1) of the code of medical ethics, no principles may be recognised and no regulations or instructions may be complied to, which are incompatible with one's tasks [64]. One pedagogical objective should therefore be to foster the development of a professional identity that can confidently distinguish its own field of decision, activity and interest from the rationale and logic of other fields, such as the logic of immigration policy. In our course, analytical processes in this direction were initiated through exchange with actors of the field and an intensive discursive exchange. Inspired by practical experiences in the refugee outpatient clinic, the seminar allowed participants in an open, non-competitive learning environment to reflect on themselves and “the” “others” [65], [66], [67] on constructions of difference, [68], [69] discrimination, [70], [71] inequality and power relations as well as on the complex structures and processes in which refugee care is embedded (cf. [72], [73]). 

Through our course, students have acquired a multi-perspective, context- and diversity-sensitive approach, relevant to their later professional everyday life, which goes beyond a mere focus on ethnic-cultural differences [74] (cf. [75], [76], [77]). To this end, we developed multiple perspectives on a complex field of topics and activities and promoted the ability to change perspectives in order to be able to develop an inner picture that is as holistic and situationally appropriate as possible. We were therefore pleased that aspects of this multi-perspectivity through multidisciplinarity were often mentioned within the take-home messages of students.

## 7. Conclusion

To prepare medical students for the medical, administrative, ethical, communicative, socio-cultural, strategic, and political challenges of health care for refugees, specific multidisciplinary teaching initiatives are needed. By means of a teaching programme, which combines theoretical, practical and reflective elements, medical students in Heidelberg were sensitised to the concerns of refugees and prepared for their care and the corresponding care contexts. In the process reflexion of the interaction of global, national and local dimension of health and care for refugees as well as the manifold interrelationships of the medical with other systems of society was initiated in order to prepare the students for their important role as advocates of health equality. The increase in knowledge through the combination of lectures, observation and reflection was rated highly. Especially through the field placement practical skills could also be acquired. The course concept can therefore serve as a basis and stimulus for the implementation of similar courses at other universities. 

## Funding

The clinical elective “Health and Medical Care for Refugees” was realized through a project cooperation between the Department of General Medicine and Health Services Research and the Heidelberg Institute of Global Health at the University Hospital of Heidelberg. The elective was financed by the Ministry of Science and Art in Baden-Württemberg (2016-2019) as part of the teaching and research project “Migration & Health”. 

## Acknowledgements

**We would like to thank the lecturers of the seminar for their commitment and willingness to share their knowledge and experience and to address student questions in their presentations, as well as their readiness to develop their classes further according to continuous evaluation:**

Dr. Janine Benson-Martin (Public Health Department, Pforzheim/Enzkreis, Heidelberg University Hospital, Institute of Global Health), Dr. Brigitte Joggerst and Angelika Edwards (Public Health Department, Pforzheim/Enzkreis), Dr. Holger Clemen, Dr. Ronny Lehmann, PD Dr. Johannes Pfeil (Heidelberg University Hospital, Centre for Children’s and Youth Medicine, Refugee Outpatient Clinic PHV), David Pfisterer (Heidelberg University Hospital, Department of General Medicine and Health Services Research, Refugee Outpatient Clinic PHV), PD Dr. med. Marija Stojkovic (Heidelberg University Hospital, Centre for Infectiology), Birgit Fuchs, Michiko Rodenberg (midwives, Refugee Outpatient Clinic PHV), Anna Hermann (Consultant for Interpreting in Health Care, German Federal Association of Interpreters and Translators, BDÜ), Prof. Dr. Verena Keck (Goethe University Frankfurt, Institute for Ethnology, Medical Anthropology Team), Dr. Julia Thiesbonenkamp-Maag (University Hospital Mannheim, Medical Anthropology Team), Natalie Manok, Prof. Dr. Christoph Nikendei, Dr. med. David Kindermann (Heidelberg University Hospital, General Internal Medicine and Psychosomatics, Refugee Outpatient Clinic PHV), Jörg Schmidt-Rohr (Association for Integration and Qualification e.V. Heidelberg), Prof. Dr. med. August Stich (Missionary Medical Clinic Hospital Würzburg, Department of Tropical Medicine, Refugee Outpatient Clinic Würzburg)

**Special thanks go to our colleagues in the refugee outpatient clinic (PHV) for their willingness to let students participate in their daily routine and share their experiences. We would especially like to thank:**

Dr. med. Steffen Kratochwill (Heidelberg University Hospital, Department of General Medicine and Health Services Research, Refugee Outpatient Clinic PHV), Sabrina Raber (Manager of Central Medical Office, Refugee Outpatient Clinic PHV)

**For good cooperation in the project and support in the start-up financing, stabilisation and implementation of the course into the Global Health Track of the Medical Faculty:**

Dr. Claudia Beiersmann, Prof. Dr. med. Albrecht Jahn (Heidelberg University Hospital Heidelberg, Institute of Global Health)

**For review of the manuscript and helpful feedback:**

Dr. Charlotte Ullrich (Heidelberg University Hospital, Department of General Medicine and Health Services Research)

**For their great commitment and reliability in supporting the organisation of the seminar as well as their open door for the students: **

Lea Stock, Tabea Mächtel, Mariella Stiller, Eilin Rast (Heidelberg University Hospital, Department of General Medicine and Health Services Research)

**For review of the English translation:**

Victoria Saint (Department of Population Medicine and Health Services Research, Bielefeld School of Public Health, Faculty of Health Sciences, University of Bielefeld)

## Competing interests

The authors declare that they have no competing interests. 

## Supplementary Material

Examples of specific student questions at the beginning of the course (moderation card enquiry, 120 individual cards)

Examples of break-out groupwork (Debriefing sessions of field placement)

Guidelines for the field report: Clinical elective "Health and medical care for asylum seekers" (Winter semester 2018/19)

Final evaluation "Health and Medical Care for Asylum Seekers”

Self-reported learning outcomes

## Figures and Tables

**Table 1:**
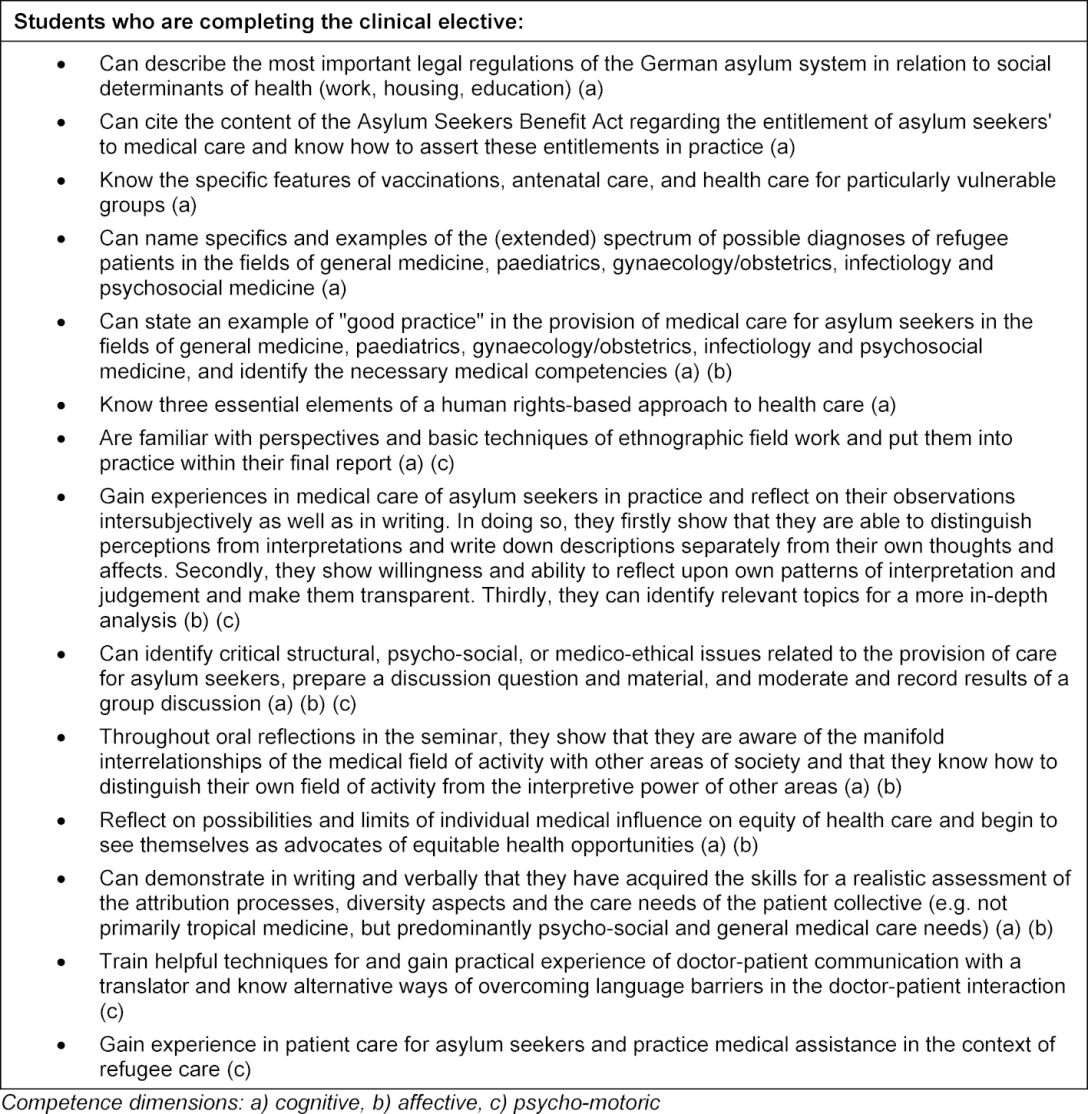
Cross-cutting learning objectives

**Table 2:**
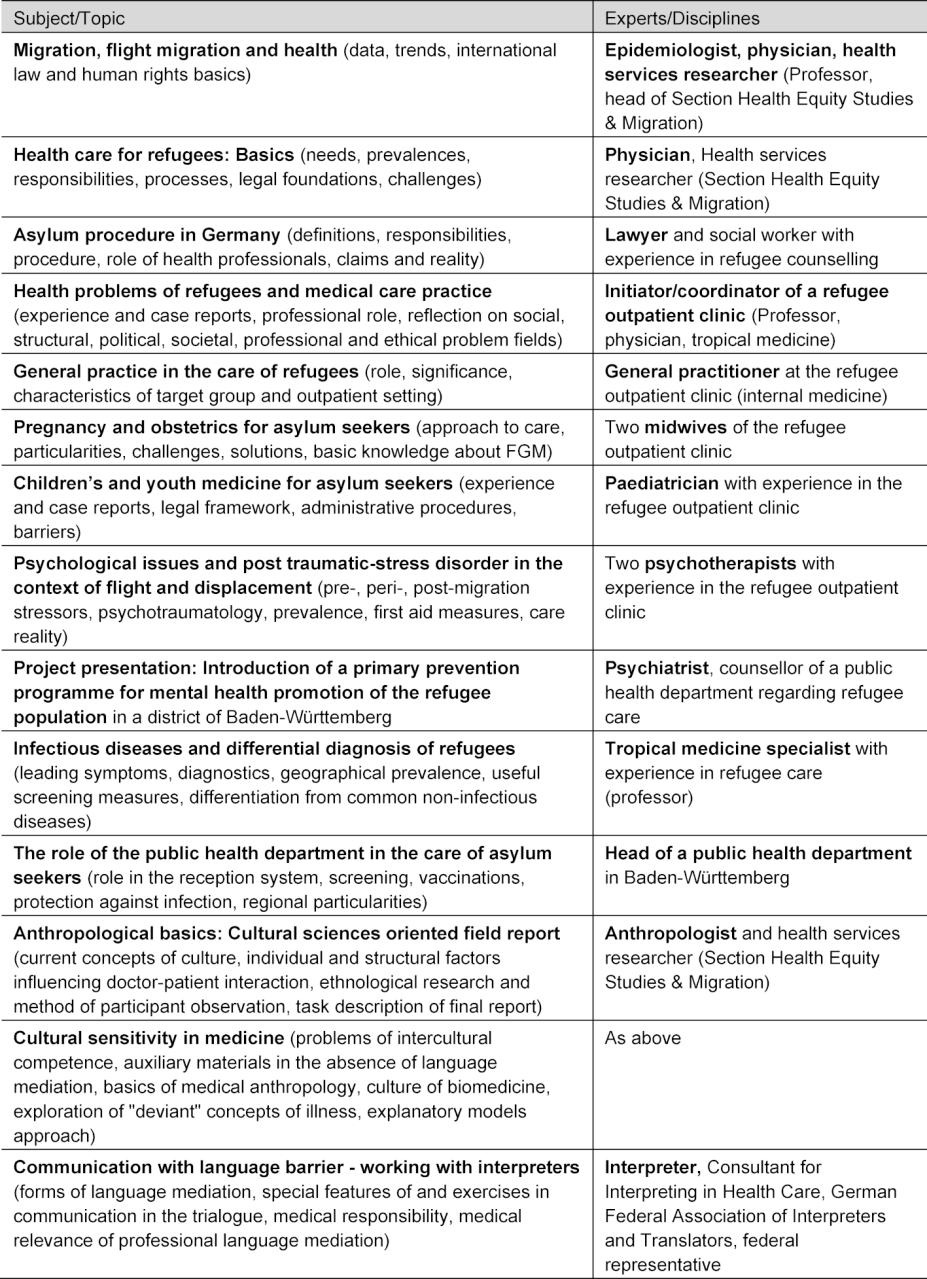
Topics and expertise of lecturers

**Table 3:**
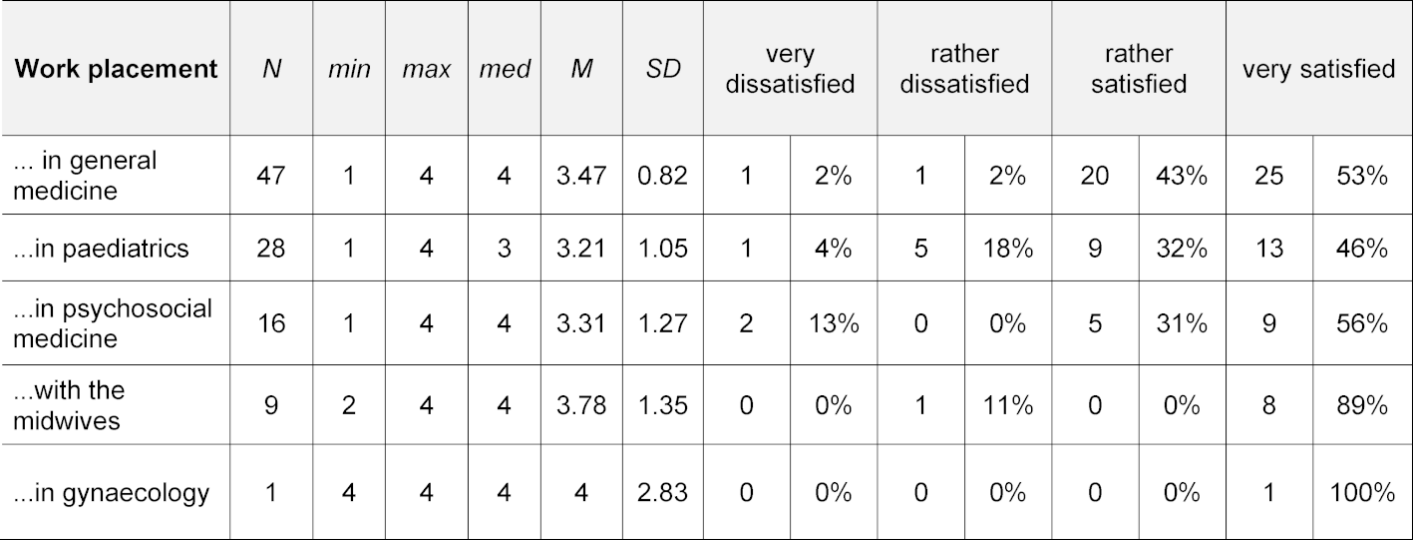
Evaluation of field placement – by medical disciplines
