# Supplementary material for: Health and medical care for refugees: design and evaluation of a multidisciplinary clinical elective for medical students
Source: GMS J Med Educ. 2021 Feb 15;38(2):Doc39. doi: 10.3205/zma001435 (PMC7958918; doi:10.3205/zma001435)
Supplement: Examples of specific student questions at the beginning of the course (moderation card enquiry, 120 individual cards) [file JME-38-2-39-s-001.pdf]

Attachment 1: Examples of specific student questions at the beginning of the course (moderation card enquiry, 120 individual cards)

Exemplary quotations from student questions on health and medical care for asylum seekers
**Challenges, differences and specificities of the patient population**
• *What new challenges will the increased influx of refugees pose for me as a doctor?*<br>• *What is the difference between medical care for asylum seekers and "traditional" medical care? (infectious diseases, psychological stress...)*<br>• *What are the things to look out for when working with asylum seekers? (legal issues, health, self protection)?*
**Asylum procedure**
• *What does the asylum procedure look like? What are the obstacles?*<br>• *What have the asylum seekers been through in terms of the "bureaucracy marathon"?*<br>• *Does the state of health play a role in the asylum procedure?*
**Legal basis of medical care, care restrictions and health system integration**
• *What are the differences between medical care for asylum seekers and German citizens?*<br>• *Are asylum seekers treated fairly? Do they receive the same care as Germans?*<br>• *How does the care of asylum seekers (structural organisation) work? Who is responsible? Who pays?*<br>• *To what extent (bandaids to emergency surgery) are asylum seekers entitled to medical care?*
**Specific administrative and legal issues**
• *What formalities do I have to consider (insurance etc.) when an asylum seeker comes to me?*<br>• *(Where) Do I, as a doctor, encounter legal problems in the care of asylum seekers (e.g. illegal status)?*
**Typical disease spectrum and expectation of an extended spectrum**
• *What are the common diseases that a PHV doctor encounters?*<br>• *What additional examination measures are required for asylum-seekers that are not necessary in general population?*<br>• *Are there additional protective measures for doctors/nursing staff?*<br>• *Are screenings for asylum seekers useful, e.g. with regard to tuberculosis, hepatitis, etc.?*<br>• *What are the epidemiological differences?*<br>• *Which, in our latitudes uncommon, disease patterns can I encounter?*
**Professional handling of mental health issues**
• *How should I deal with asylum seekers who have suffered severe psychological trauma during their flight?*<br>• *Which psychological counselling centres/psychiatric care units can I refer refugees to?*<br>• *How does psychotherapy work despite language difficulties and high mobility of refugees?*
**Linguistic understanding and conditions for successful doctor-patient communication**
• *What should you pay attention to in the communication?*<br>• *What should you pay attention to in a conversation? Should one discuss the topic "migration", "home", "homesickness"?*<br>• *How can the language barrier be overcome?*<br>• *Who can and may interpret quickly and unbureaucratically and interprets well? Where can I find the interpreters?*<br>• *What do you do if you do not have an interpreter available?*<br>• *How do I manage to communicate with patients and build a bond with them despite the presence of interpreters on one level?*

Attachment 1 to: Ziegler S, Wahedi K, Stiller M, Jahn R, Straßner C, Schwill S, Bozorgmehr K. *Health and medical care for refugees: design and evaluation of a multidisciplinary clinical elective for medical students.* GMS J Med Educ. 2021;38(2):Doc39. DOI: 10.3205/001435

**Expectations of and dealing with cultural difference**
• *What might I not understand?*<br>• *How do I deal with exceptional situations (e.g. difficult communication and religious/cultural aspects)?*<br>• *How do I deal with different models of health, illness and the role of a doctor?*<br>• *What do refugees expect from healthcare in Europe? How do they want to be treated?*<br>• *How do I deal with a patient refusing treatment because of cultural differences/specific beliefs?*<br>• *How do I deal with patients from other cultures (e.g. male doctor - women with hijabs or female doctor - muslim man) and with relatives?*
