# Supplementary material for: Health and medical care for refugees: design and evaluation of a multidisciplinary clinical elective for medical students
Source: GMS J Med Educ. 2021 Feb 15;38(2):Doc39. doi: 10.3205/zma001435 (PMC7958918; doi:10.3205/zma001435)
Supplement: Examples of break-out groupwork (Debriefing sessions of field placement) [file JME-38-2-39-s-002.pdf]

Attachment 2: Examples of break-out groupwork (Debriefing sessions of field placement)

Objective: Reflection on the social context as well as the structures and processes in which medical care of asylum seekers is embedded.

Task:

After getting to know the daily routines in the outpatient clinic of the arrival and registration centre for asylum seekers, each student has to prepare one of the following things to be worked on in a small group of fellow students (see guidelines, Annex 3, pt. 4):

- Casework, experienced situation → collective reflexion from multiple perspectives
- Preparing a critical question and moderating the discussion
- Selecting a scientific or journalistic text to be read and discussed with fellow students
- Showing parts of the preparation for the final report and discussing its content or methodology

Examples of topics from group discussions (for illustrative purposes – three described in detail, others briefly noted)

- M. reports in the working group on a patient who had mentioned that he could not buy the nasal spray recommended by the doctor himself due to lack of money. M. brought the legal basis for living expenses and pocket money for asylum seekers to the debriefing session and talked to her fellow students about what was part of their own daily needs and whether the amount was sufficient. Arguments for and against the exclusive handing out of material resources were collected and the possibilities and limits of issuing vouchers were discussed.

- K. presents a case in which a patient was sent to the psychologist at the outpatient clinic on the recommendation of a social worker to ask for a certificate regarding the lack of fitness to travel. According to the doctor, the patient was "psychologically burdened but medically unimpaired", so that in his opinion a certificate would not have the desired influence on the process. K. identified here the issues she wanted to discuss with her fellow students: false/disappointed hopes, medical certificates, possible intransparency and misinformation. Therefore, she formulated a thesis: "A considerable part of the suffering associated with the asylum procedure is caused by intransparency and misinformation". She had also printed out the "Merkblatt für die Überprüfung der Reisefähigkeit vollziehbar ausreisepflichtiger Ausländer" (leaflet for the examination of the ability to travel for foreigners who are obliged to leave the country), which was read together. The students exchanged their experiences in the refugee outpatient clinic with regard to patient requests for medical certificates and discussed, among other things, the situations of the doctor. They reflected among other things on whether the medical care process might suffer under expectations and requirements like that. Furthermore, they speculated whether intransparency, e.g. regarding the criteria for legally valid expert opinions and certificates, might be politically desired. The group also noted that the availability of counselling services on the asylum process and medical care may vary from region to region, and that the level of knowledge and experience of the providers consulted may also differ, which inequality of opportunities for asylum seekers.

- In another small group, a student raised the – somewhat related – question of whether "neutral treatment" is possible in this context and whether one does not already exert influence by issuing or not issuing a certificate. The group discusses whether it was okay to impose a responsibility on the physician that affects not only the health but the whole life of the patient. The students asked themselves in which cases own “political” interference is allowed and necessary and where a line should be drawn.

Other topics discussed in workshop groups:

- Dealing with the (for patients) shameful topic of Female Genital Mutilation/Circumcision (exchange of practical experience, workshop on doctor-patient communication)
- Sharing of the oppressive feeling at the security check at the entrance to the former barracks and the walk through the fenced area, which is remote from the city (printed newspaper clippings were used to discuss the choice of this and similar forms of accommodation; students considered the effect of the type and location of accommodation on the "inmates" and staff as well as on integration efforts)
- Analysis of the nature of the parallel care setting of the refugee outpatient clinic as a hitherto unknown setting (identification of institutional and organisational problems of the outpatient clinic, e.g. different documentation systems, high number of doctors, changing responsibilities, lack of time, lack of communication between individual disciplines, availability of materials)
- Reflection on how to deal with benzodiazepine dependence and addiction in the PHV (exchange of experiences, identification of challenges, workshop on possible improvements and solutions)
- Dealing with expressed suicide intentions in refugee settings (exchange of experience, collection of possible best practice examples)
- Discussion of structural problems that make care more difficult (case report: psychotic Syrian patients for whom the transfer to the follow-up accommodation was repeatedly postponed, which delayed the start of necessary psychotherapeutic treatment, discussion of possible medical action strategies)
- Reflection on the role of doctors in cases of denied benefits (collection of experience reports on disappointed patients who were denied medical services because they were not paid for by the funding agency (analysis of the § 4 and § 6 AsylbLG, discussion on their different "interpretability")
- Short presentation on the suspension of family reunification for persons with subsidiary protection status (critical discussion on this decision, discussion on consequences for families and integration)
- Reflection on the understanding of the medical professional role (by means of reading: a media report on doctors who refused to treat refugees, a section from the professional code of conduct and the legal text “non-assistance” – discussion on the fulfilment of the conditions for this offense)
- Discussion of the topic of Health-Seeking-Migration (based on a newspaper article and the question whether it makes sense to identify an independent group here or if we talk about people fleeing from countries with precarious political or economic conditions where medical care does not meet our standards)

Following the group discussions,

- the groups reported on their discussed topics and results in the plenary
- each student recorded his or her process of identifying a topic, preparation and the discourse process in the group in their final report.

There has been lively work in all groups. Often no consensus was reached; instead, knowledge, experience, and perspectives were gathered. Many discussions did not result in a final answer, but in more questions being raised and new topics to be reflected on. We not only accept this, but consciously foster it, in order to stimulate individual and collective cognitive reflection processes, which may lead to a deeper examination and understanding of aspects that go far beyond a focus on the body of an asylum seeker.
