# Supplementary material for: Health and medical care for refugees: design and evaluation of a multidisciplinary clinical elective for medical students
Source: GMS J Med Educ. 2021 Feb 15;38(2):Doc39. doi: 10.3205/zma001435 (PMC7958918; doi:10.3205/zma001435)
Supplement: Guidelines for the field report: Clinical elective "Health and medical care for asylum seekers" (Winter semester 2018/19) [file JME-38-2-39-s-003.pdf]

Attachment 3: Guidelines for the field report: Clinical elective "Health and medical care for asylum seekers" (Winter semester 2018/19)

Field Report

Formalities and data protection

- □ Cover sheet with name, matriculation number, semester, visited disciplines
- □ Scope: approx. 8-15 pages, insert page numbers!
- □ Do not collect personal data (names of doctor, patient, parties involved are changed or exchanged for letters)

1. Field placement report (approx. 5 pages)

1.1 Process description: First describe the organisational process in the Patrick-Henry-Village from patient admission until the patient leaves the clinic (can be done in bullet point form)

1.2 Non-participatory observation: Take about 30 minutes and observe a consultation without an interpreter (inform the physician you are working with that you will stay passive while observing). Write up your field notes according to the methodological instructions in the course as well as the notes and table on p. 2.

2. Reflection (approx. 2-3 pages)

2.1 What did you find particularly interesting during your field placement? Specify two points in more detail:

2.1.1 a medical aspect e.g. anamnesis, diagnosis, diseases

2.1.2 a structural, socio-cultural or psycho-social aspect (e.g. thoughts on doctor-patient interaction, context of the protagonists, possible influence of structural factors, habits that are different from your own, influence of housing situation, organisational and legal framework etc.)

2.2 Is there a situation in which you would have acted differently than the colleague you observed? How would you have acted in practice and why?

2.3 Is there a situation that you think the colleague has mastered very well? Which competencies could you identify?

3. Self-reflection - Learning objectives - Open questions (approx. ½ page)

3.1 Which aspects/issues would you like to know more about? In your opinion, what aspects of the work with asylum seekers/refugees that you encountered during your time in the outpatient clinic have you not yet been prepared for in your medical studies? (can be done in bullet point form)

3.2 What learning objectives have you identified for yourself? What do you want to find out or train and how are you going to do it? (present concrete strategies for competence acquisition)

Attachment 3 to: Ziegler S, Wahedi K, Stiller M, Jahn R, Straßner C, Schwill S, Bozorgmehr K. *Health and medical care for refugees: design and evaluation of a multidisciplinary clinical elective for medical students.* GMS J Med Educ. 2021;38(2):Doc39. DOI: 10.3205/001435

4. Debriefing of the field placement (approx. ½ - 1 page)

4.1 Case report/supervision/discussion:
Think about what aspect of your observation/question or case you would like to discuss with the seminar group.
Present your ideas in a small group in the seminar (debriefings) e.g.
- in the form of a question to be discussed,
- the joint reading of a part of the report,
- of a scientific or journalistic article,
- of a role play,
- a task for a group work

(Please attach your prepared material to the report!)

4.2 Short Prototoll:
Report on your discussion/group work and its results on the last page of your report.

Observation aids (regarding point 1.2. of the report)

What should I observe and describe? Procedure for non-participatory observation

1. Framing conditions: Place, date, time of day, persons involved, ...
2. Inform doctor and patient / ask for permission (that you as a medical student take notes for a course an that no names and identifying details are noted)
3. Field notes: observe, make handwritten notes
4. Finish field notes: Leave the situation, add notes immediately after observation. Write down questions, personal impressions/feelings, and considered first interpretations
5. Create a protocol: Look through the material at home, digitalize it (in table form), consider further interpretation possibilities and add analytical notes
6. Consider subheadings e.g. history, anamnesis, getting to know the patient, clarifying misunderstandings, physical examination, documentation etc.
7. Identify topics (in column 3): What is at stake here? What overarching topic do I see? e.g. communication difficulties due to lack of language skills, structural conditions (housing situation, access barriers); different perception of the problem by the doctor/patient; what is the relationship between this observation and the larger context (e.g. what legal requirements, institutional regulations play a role, etc.) -> You can come back to some of the aspects here in the reflection-part (point 3) of the report
8. Heading: Find a heading for the whole observation

What do I write in my report? Structuring of writing (scheme for point 2)

**Observation/description** (1)	**Analytical Notes**: Open questions/ notes/thoughts/affects/interpretations (2)	Identified **categories/** general terms/themes (3)

Possibly in landscape format, column (3) refers mainly to column (1)

Attachment 3 to: Ziegler S, Wahedi K, Stiller M, Jahn R, Straßner C, Schwill S, Bozorgmehr K. *Health and medical care for refugees: design and evaluation of a multidisciplinary clinical elective for medical students.* GMS J Med Educ. 2021;38(2):Doc39. DOI: 10.3205/001435

<u>What do I describe? Focus and observation tips</u>

- □ **Equipment, furnishing** and their influence on the consultation, material and equipment which is used/relevant for the situation
  → *Do not take anything for granted, decide on the scope yourself, imaging a reader that is not familiar with the situation and its background*
- □ **Time, duration, first/following treatment?**
- □ **Participants**, description of the participants
  → Sex, estimated age, country of origin, posture, clothing, mode of communication (language proficiency of doctor and patient)
  →*Attention - describe!* *The man is wearing torn, dirty jeans.*
  NOT: The man looks poor or scruffy.
- □ **Sequences of interaction** (chronological)
- □ Describing behaviour and interaction
  → *Do not interpret:* *The woman cried and hit her head with her hand.*
  NOT: The woman is traumatised/insane.
- □ Describing **posture, facial expressions, gestures**
- □ **Content of the communication** (Who says what? Record as precisely as possible: also use quotations).
  → Special attention to: reason for consultation, medical history (start, course, what information does the patient give on his/her own?) What is the most troublesome aspect to him/her? How does he/she imagine the therapy? What questions does the doctor ask? What aspects does he focus on? What explanations does he/she give about the illness/therapy? What does the patient think about the therapy? What questions does he/she ask)?
- □ **Style of communication**: speed and speaker changes, volume and intonation, use of non-verbal communication/sign language (What signs are used?).

Focusing→ Let yourself be guided by the relevance of the participants: What do they see? What is on their minds? What do they talk about? What practical problems do they have?

For more literature as well as an example of an observation protocol (Scheffer, Thomas: Asylgewährung. An ethnographic process analysis. Stuttgart 2001) see electronic course room
