# Supplementary material for: Health and medical care for refugees: design and evaluation of a multidisciplinary clinical elective for medical students
Source: GMS J Med Educ. 2021 Feb 15;38(2):Doc39. doi: 10.3205/zma001435 (PMC7958918; doi:10.3205/zma001435)
Supplement: Final evaluation "Health and Medical Care for Asylum Seekers” [file JME-38-2-39-s-004.pdf]

Attachment 4: Final evaluation
"Health and Medical Care for Asylum Seekers"

How did you find out about the seminar?				
Take-home-message. What did you take with you?<br>What was the most important thing you learned in the course? What was new to you or surprised you? And why was this learning content so important or impressive for you?				
How strongly do you agree with the statement				
	fully disagree	rather disagree	rather agree	fully agree
The participation in the elective was worthwhile for me				

Attachment 4 to: Ziegler S, Wahedi K, Stiller M, Jahn R, Straßner C, Schwill S, Bozorgmehr K. *Health and medical care for refugees: design and evaluation of a multidisciplinary clinical elective for medical students.* GMS J Med Educ. 2021;38(2):Doc39. DOI: 10.3205/001435

**Your evaluation of the learning content** *(*greyed out areas are not covered in the GMS manuscript 2021)*<br>Please assess the basic relevance of the contents for medical education:<br>**1: Highly relevant** - should be implemented into the standard medical curriculum<br>**2: Relevant** - should continue to be taught in the context of the compulsory elective<br>**3: Not very relevant** - could be made available to students with special interests, e.g. through literature references or internships<br>**4: Not relevant** - can be removed from the curriculum	
Subject	Relevance of the topic
Flight and migration in numbers (migration routes, global connections)	
Legal aspects and procedures of the asylum procedure	
Legal bases and processes of medical care	
The role of public health departments in the care of asylum seekers	
Old and current definition(s) of culture	
Understanding and applying the anthropological method of observation	
Interpreting in the medical field and working with interpreters	
Infectious diseases and differential diagnosis of refugees	
Getting to know medical care for asylum seekers in the PHV	
Pregnancy care and obstetrics for asylum seekers by midwives	
Traumatisation and psychological stress in refugees and asylum seekers	
General medical care (family doctors/PHV)	
Culture- and context-sensitive doctor-patient communication	
Children´s and youth medicine for asylum seekers	

If you want to justify or comment on your relevance rating, please do so here:
What other **content** should be included in the **curriculum**?

Your evaluation of the field placement

Please tell us how satisfied you were with your placement.

With the work placement in…	Very dissatisfied	Rather dissatisfied	Rather satisfied	Very satisfied
...general medicine				
...paediatrics				
...psychosocial care				
...midwifery				
…gynocology*				
o I did not participate in the field placement				

Attachment 4 to: Ziegler S, Wahedi K, Stiller M, Jahn R, Straßner C, Schwill S, Bozorgmehr K. *Health and medical care for refugees: design and evaluation of a multidisciplinary clinical elective for medical students.* GMS J Med Educ. 2021;38(2):Doc39. DOI: 10.3205/001435

Your notes on the field placement<br>Do you have any comments or suggestions regarding the field placement, suggestions for the course team or other things you would like to tell us about the internship?

**Evaluation of your competence acquisition through the seminar components:**					
	**does not apply**	**does rather not apply**	**partially applies***	**rather applies**	**applies**
By participating in the course I expanded my knowledge regarding health care of asylum seekers.					
By participating in the course I expanded my practical skills regarding health care for asylum seekers.					
My field placement at the Patrick Henry Village led to an expansion of knowledge of health care for asylum seekers					
My field placement at the Patrick Henry Village led to an expansion of my practical skills in the field of health care for asylum seekers					
By verbally reflecting on my experiences during the field placement with my fellow students I expanded my skills					
Through the written reflection of my experiences during the field placement I was able to expand my competences					
*Translation from the german: „teils-teils" meaning partly applies, partly does not apply					

Do you have any comments on the ethnographic observation and the final report?

What I **liked** about the seminar:

Attachment 4 to: Ziegler S, Wahedi K, Stiller M, Jahn R, Straßner C, Schwill S, Bozorgmehr K. *Health and medical care for refugees: design and evaluation of a multidisciplinary clinical elective for medical students.* GMS J Med Educ. 2021;38(2):Doc39. DOI: 10.3205/001435

What I did **not like** about the seminar:

My tip to the organisers for the future design of the course:

Please mark the seminar with a school grade (1-6): I give the elective the grade ☐

Thank you very much for your feedback! ***Your course team***

Attachment 4 to: Ziegler S, Wahedi K, Stiller M, Jahn R, Straßner C, Schwill S, Bozorgmehr K. *Health and medical care for refugees: design and evaluation of a multidisciplinary clinical elective for medical students.* GMS J Med Educ. 2021;38(2):Doc39. DOI: 10.3205/001435
