# Supplementary material for: Health and medical care for refugees: design and evaluation of a multidisciplinary clinical elective for medical students
Source: GMS J Med Educ. 2021 Feb 15;38(2):Doc39. doi: 10.3205/zma001435 (PMC7958918; doi:10.3205/zma001435)
Supplement: Self-reported learning outcomes [file JME-38-2-39-s-005.pdf]

Attachment 5: *Self-reported learning outcomes*

Example answers to the evaluation question: “Take-home-message. What did you take with you? What was the most important thing you learned in the course? What was new to you or surprised you? And why was this learning content so important or impressive for you?” (Citation of repeatedly stated content)

**Insights gained from practical experience (29 individual references)**
Practical experience as most important learning experience
• *I got the most out of the field placement. The direct contact with the refugees is simply a longer lasting impression than statistics, slides etc... Much of what I learned in the seminars - at least that's how I feel - is quickly forgotten, but the direct confrontation is what invites you to reflect and act accordingly. (WiSe2017/18)*<br>• *Most important were the field experiences. Insights into the care process, patient experiences and the local situation were very impressive and moving experiences, which also sensitised me for my future medical profession. [...] (WiSe2017/18)*<br>• *For me, the observation was probably the greatest enrichment. This personal impression is invaluable. It has inspired me to many new thoughts and which I have discussed with many people in my environment. (SoSe2018)*
Getting to know individual fates and perspectives of refugees -> leading to individualisation
• *The most important thing for me was learn about the personal "fates" of patients/refugees [...]*<br>• *Getting an impression of the health seeking behaviour of refugees and what they are like (SoSe 2018)*<br>• *For me it was especially important to feel how the patients feel in the PHV. I think I have gained some insight on it. Without the elective, I would not have been able to obtain this emotional knowledge. (SoSe2017)*<br>• *During my field placement in the PHV I learned that as a doctor it is very important to look at each patient individually and to take them seriously, even if it is difficult to devote the same amount of compassion and empathy to each patient. (SoSe 2018)*<br>• *Structural obstacles should be tackled, but it is equally important as a doctor to see the person and respond to them individually (despite cultural/social/etc. differences). The important thing is to try. [...] in the end what counts is an authentic and accepting interpersonal relationship. [...] (WiSe2017/18)*
Surprises: Lack of a centralised structure, care restrictions, dependence on individual engagement/improvisation
• *The PHV relies on many individual committed employees, the mission support from the political/state level is rather disappointing. (SoSe2017)*<br>• *I am negatively surprised how badly communication, processes and responsibilities are organised by the state. (SoSe2018)*<br>• *How little of the medical care is covered was not unexpected but nevertheless surprising to me. (SoSe2017)*<br>• *I was surprised to see that the whole health care team is still made up of so many volunteers and private organisations, in spite of the decreased influx. (SoSe2017)*<br>• *It was very impressive to learn that all actors involved (government officials, doctors, PHV, social workers, volunteers, ...) deal with the problems and challenges in a more or less "improvised" way. [...] (SoSe2017)*
Expected unfamiliarity - surprising ordinariness
• *What was particularly surprising was that in the PHV (for the experts that deal with asylum seekers), the same problems occur as in a practice or a small hospital and the different solution strategies do not always work. (SoSe 2018)*<br>• *That asylum seekers have the same problems as we locals. It shows that they are not "second-class people". I was very surprised at how ordinary the problems were that asylum seekers had when they went to see a doctor. [...] (WiSe2017/18)*<br>• *The people who come to Germany now are no different from the everyday patient! [...] (SoSe 2018)*<br>• *[...] the diseases the refugees have are mostly diseases that they have caught in Germany, especially among children [...] (SoSe 2018)*

Theory-Practice-Transfer

- *I very much enjoyed the field time. I was able to view much of the course content (infections, accommodation, midwifery) directly, thus deepening what I had learned and recognising its importance. (WiSe 2017/18)*
- *I was also very surprised by the interpreter's presentation, as it highlighted many aspects that one does not think about at first. While sitting in on consultations, one could see these aspects in practice or look for them in the local translators practices. (WiSe 2017/18)*
- *I have developed a feeling for the problems of health care for asylum seekers, I now know better what is at stake. [...] In the elective, subjective opinions [of refugees, doctors, lecturers] could be dealt with in an objective framework - this way one could better understand the actual relevance of theoretical questions and possibly form one's own opinion. (SoSe 2018)*

Learner centricity and multi-perspectivity through oral reflection

- *As a prospective doctor, the field placement in the PHV was very valuable for me - also including the subsequent discussion rounds in the context of the elective (discussion of open questions and general problems). (winter term 2017/18)*
- *With regard to [the field placement], the exchange with the other students was of course useful and interesting (sometimes the doctors/their behaviour was perceived quite differently). [...] (SoSe 2017/18)*

Legal bases concerning the asylum procedure (15-20 individual references)

- *Most helpful was the presentation on asylum procedure/asylum law -> extensive and difficult to grasp. [...] (WiSe2017/18)*
- *Legal bases on asylum procedures and medical care: particularly important and new to me. (...) (WiSe2017/18)*
- *Most important: The 4 different statuses an asylum seeker can have after a successful asylum application. (SoSe2017)*
- *(...)the laws/bureaucracy is dry, but interesting and necessary to understand the background. (WiSe2017/18)*
- *The different ways in which a law can be interpreted and implemented. (SoSe 2018)*

Multiperspectivity through multidisciplinarity (10-15 individual references)

- *Different perspectives on refugee issues. [...] (SoSe2017)*
- *The learning content was impressive because the topic was not only approached from the medical side, but many other subjects were also included. (WiSe2017/18)*
- *[...] almost holistic presentation (in the elective) of the problem areas in the care of refugees (WiSe 2017/18)*
- *I have learned how many different aspects of the care of asylum seekers need to be considered in order to get a complete picture. (SoSe2017)*

Overarching relationships

- *I now pay more attention to patients and am more aware that laws and geopolitical situations not only affect the health of the population, but also the work of doctors. (SoSe2017)*
- *I have learned how much responsibility the medical profession has, not only medically but also politically. One moves in a complex field of tension. (SoSe2018)*

Generation of new questions

- *I have the feeling that I have at least got a good overview of a number of topics during the elective. When I look at the questions we wrote down at the beginning, it seems to me that we have answered most of them, but this is an overview. Since then, I'm sure just as many questions have arisen (Sunse 2018).*

Insight into the subject matter, away from media presentation, factual knowledge (approx. 6 individual references)
• *It was very interesting to see how much one's own image on a topic is influenced by the media, which expresses more appeals than allows insights. And there no factual information is provided (SoSe2018)*<br>• *[...] more background knowledge/understanding for discussions on the topic of migration/asylum" (SoSe2018)*<br>• *[...] significant increase in knowledge of the hard facts of care (SoSe2018)*

Learning progress on specific seminar topics (example quotations of the topics with 2-4 four individual references)
Differential diagnosis/extended disease spectrum:<br>• *African Dermatology (Nothing learned in medical training) 2. Metabolic differences in different ethnic groups (Nothing learned in medical training) 3. Visual diagnosis (Too little practised in medical training) (SoSe2017)*<br><br>*Cultural theory/cultural difference*<br>• *The cultural differences in subjective explanatory models of disease, dealing with diseases, nutrition etc. (SoSe 2018)*<br>• *The topic of culture, especially what is "culture" (SoSe 2018)*<br>*Language mediation*<br>• *I was surprised by the interpreter-problem. I was not aware of the difficulties there. (SoSe2017)*
